# Swertisin an Anti-Diabetic Compound Facilitate Islet Neogenesis from Pancreatic Stem/Progenitor Cells *via* p-38 MAP Kinase-SMAD Pathway: An *In-Vitro* and *In-Vivo* Study

**DOI:** 10.1371/journal.pone.0128244

**Published:** 2015-06-05

**Authors:** Nidheesh Dadheech, Abhay Srivastava, Neha Paranjape, Shivika Gupta, Arpita Dave, Girish M. Shah, Ramesh R. Bhonde, Sarita Gupta

**Affiliations:** 1 Molecular Endocrinology and Stem Cell Research Lab, Department of Biochemistry, Faculty of Science, The M S University of Baroda, Vadodara, Gujarat, India; 2 Hislope College of Biotechnology, Nagpur, Maharashtra, India; 3 Skin Cancer Research Laboratory, Centre de Recherche du CHUL, CHUQ, Univerisity Laval, Quebec City, Quebec, Canada; 4 Manipal Hospital and Regenerative Medicine Centre, Manipal Hospital, Manipal, Karnataka, India; CHA University, REPUBLIC OF KOREA

## Abstract

Transplanting islets serves best option for restoring lost beta cell mass and function. Small bio-chemical agents do have the potential to generate new islets mass, however lack of understanding about mechanistic action of these small molecules eventually restricts their use in cell-based therapies for diabetes. We recently reported “Swertisin” as a novel islet differentiation inducer, generating new beta cells mass more effectively. Henceforth, in the present study we attempted to investigate the molecular signals that Swertisin generate for promoting differentiation of pancreatic progenitors into islet cells. To begin with, both human pancreatic progenitors (PANC-1 cells) and primary cultured mouse intra-islet progenitor cells (mIPC) were used and tested for Swertisin induced islet neogenesis mechanism, by monitoring immunoblot profile of key transcription factors in time dependent manner. We observed Swertisin follow Activin-A mediated MEPK-TKK pathway involving role of p38 MAPK via activating Neurogenin-3 (Ngn-3) and Smad Proteins cascade. This MAP Kinase intervention in differentiation of cells was confirmed using strong pharmacological inhibitor of p38 MAPK (SB203580), which effectively abrogated this process. We further confirmed this mechanism in-vivo in partial pancreatectomised (PPx) mice model, where we could show Swertisin exerted potential increase in insulin transcript levels with persistent down-regulation of progenitor markers like Nestin, Ngn-3 and Pancreatic Duodenal Homeobox Gene-1 (PDX-1) expression, within three days post PPx. With detailed molecular investigations here in, we first time report the molecular mode of action of Swertisin for islet neogenesis mediated through MAP Kinase (MEPK-TKK) pathway involving Ngn-3 and Smad transcriptional regulation. These findings held importance for developing Swertisin as potent pharmacological drug candidate for effective and endogenous differentiation of islets in cell based therapy for diabetes.

## Introduction

Islet Neogenesis refers to generation of new β-cells from progenitor cells. Insulin producing β-cells form bulk of islets (65–80%), are targeted for destruction at early stage in type I diabetes and at an advanced stage in type II diabetes. Hence, identification of novel differentiation inducer is a prime requisite for islet generation and increasing beta cell mass, which could be next generation therapeutics for diabetes. Also, there is need to understand molecular mechanism involved in β-cells differentiation using small molecule as differentiating agents. This can be exemplified by phenomenon “Ontology recapitulates phylogeny” [1]. In 2004, Melton’s group conducted an elegant lineage tracing experiment to strongly argue that pre-existing terminally differentiated β-cells retain a strong proliferative capacity *in vivo* and they are the major source of new β-cells during adult life and after partial pancreatectomy in mice [2]. Their study challenged the notion that adult pluripotent stem cells could have a significant role in β-cells replenishment [3]. In parallel, Xu et al. produced equally strong evidence that new β-cells can be generated in injured pancreas of adult mouse from its endogenous (pancreatic) progenitor/stem cells [4]. Various distinct mechanisms are postulated to account for β-cells regeneration, mainly (i) trans-differentiation of exocrine cells into endocrine β-cells; (ii) emergence of new β-cells from pancreatic ductal epithelium; and (iii) replication of pre-existing β-cells and lastly (iv) stem cell differentiation from various tissue sources [5].

To expedite the process of *in-vitro* islet neogenesis from various types of progenitor cells, we need to have a better understanding of different factors and their mode of action that can influence this process. Many studies have focused on the role of small peptides, cytokines and proteins in stem cell differentiation to obtain insulin-producing cells [6]. Some of the compounds have been instrumental in islet differentiation protocols, such as Hepatocyte Growth Factor, Insulin like Growth Factor, Activin-A, Exendin-4, Glucagon Like Peptide-1, INGAP and Betacellulin etc. Most importantly, with all above experimental evidences only two molecules Activin-A and Keratinocyte growth factor (KGF) has been explored for their mechanism of action for differentiation, till date [7, 8]. Activin-A promotes islet differentiation via ACT-MEPK-TKK pathway mediated through activin (ACT-III) receptors that drive increased phosphorylation of p38 leading to activation of Ngn-3, controlling endocrine transcriptional machinery via smad proteins for islet generation [9]. Movassat et al., demonstrated that KGF promotes beta-cell regeneration by stimulating duct cell proliferation *in vivo* by directly inducing the expression of PDX1 in some ductal cells thus leading to beta-cell neogenesis. The molecular mechanism of KGF involved direct effects on duct cell proliferation, mediated by the MEK-ERK1/2 pathway, while differentiation by regulating PI3K/AKT pathway [7]. It is pertinent to note that in both the studies MAP Kinase pathway was actively involved and responsible for differentiation.

Apart from biological or chemical inducers, very few investigators have used herbal products as known differentiating agents to obtain insulin-producing cells. One such study by Kojima et al., first time reported and introduced herbal agent named “conophylline” which showed generous differentiation of pancreatic acinar AR42J cells into insulin producing cells with elevated expression of Pdx-1, Ngn3 and GLUT-2 in differentiated clusters [10]. This group has provided few superficial evidences on differentiation mechanism of conophylline; mimicking Activin-A triggered differentiation signals *via* p38 MAP Kinase phosphorylation involving Ngn-3 up-regulation [9]. With all above reports, it is clearly highlighted that both activin-A and MAP Kinases are effectively involved in beta cell development. Therefore, for effective translational therapeutics, it becomes more important now to understand the mechanistic action of these small molecules and develop them as potential candidate for islet neogenesis.

In last few years, our lab has examined anti-diabetic, hypolipidaemic, and islet protective activity of *Enicostemma littorale* [11–13]. The plant has also been examined for its capacity in promoting islet neogenesis. Recently, we reported *in-vitro* formation of functional islet-like cell clusters containing both β and α cells from Panc-1 cells and mouse embryonic fibroblast NIH3T3 cells using small biomolecule “Swertisin” isolated from *E*. *littorale* [14, 15], as a potential molecule for beta cell generation. This led us to investigate the mechanistic action of this potent bioactive agent for islet cell differentiation property. We therefore attempted a systematic and time dependent study of transcriptional machinery involved in islet differentiation induced by Swertisin under both *in-vitro* and *in-vivo* conditions. Activin-A was used as control to compare the MEPK-TKK signal pathway during differentiation.

## Results

In order to understand the mechanism of islet neogenesis mediated by *E*. *littorale* active molecule “Swertisin”, we carried out time dependent monitoring of key transcriptional factors and progenitor markers implicated in islet formation using human pancreatic adenocarcinoma cells PANC-1 and mouse intra-islet progenitor cells (mIPC) along with Activin-A as control.

### PANC-1 cells differentiate into ILCC with Activin-A and Swertisin

**Fig 1:**
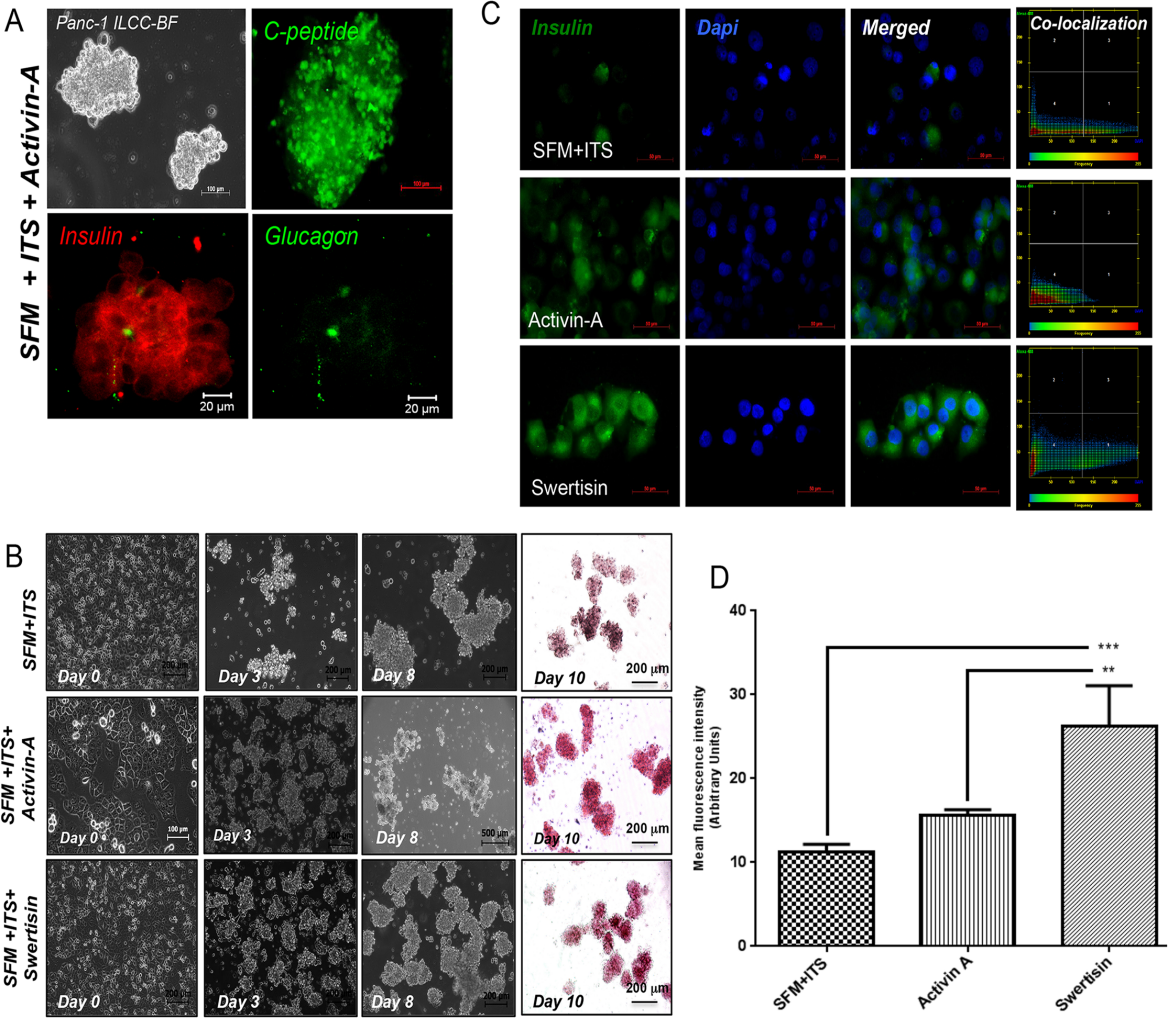
PANC-1 cells differentiation and characterization with Activin-A. (A) showed differentiation of Panc-1 cells subjected to differentiation using activin-A for 10 days. Figure shows bright field image of ILCC generated upon differentiation on day 10^th^ at 10X magnification. Panels (A) showed Panc-1 ILCC immunostained on 10^th^ day, positive for C-peptide, Insulin, and glucagon. Insulin was stained with TRITC labeled antibody showed red in color whereas c-peptide and glucagon were stained using FITC labeled antibodies showed green in color. (B) shows comparison of PANC-1 cells differentiation with Control SFM, Activin-A and Swertisin. Panc-1 cells cultured in complete media at day 0 which were then subjected to differentiation using SFM/ITS, Activin-A and Swertisin for 10 days. Bright field image of cells under differentiation for day 3^rd^, day 8^th^ at 5X and 10X magnification respectively and dithizone stained ILCC on day 10^th^ are shown. (C) shows comparative and qualitative insulin immunofluorescence signals in ILCC differentiated from SFM/ITS, Activin-A and Swertisin. Insulin is stained with Alexa-488 labeled antibody showed in red color and nucleus were counterstained with DAPI in blue. Pixel colocalization graphs shows co-localized distribution pattern of insulin and dapi fluorescence in differentiated ILCCs. (D) represents graph for quantification of insulin immunofluorescence per unit cytoplasm from immunostained ILCCs in Control SFM, Activin-A and Swertisin groups. Insulin signal stained with alexa-488 in Swertisin group compared to both SFM and activin-A group. Data was calculated from every single cell in three different frames per slide and expressed as mean ± SEM. *** and ** shows p value less than 0.001 and 0.01 in Swertisin group with respect to SFM and Activin-A group.

Human pancreatic progenitor is best system to understand the molecular mechanism of islet neogenesis and Panc-1 cells serves a simplest model for study, as there is no other model available for human progenitors. Panc-1 recently demonstrated to present various progenitor markers like C-kit and Stem cell factor (SCF) making Panc-1 as best suitable model for progenitor studies [16]. We conducted Panc-1 differentiation into islet like clusters with earlier described protocol [14, 15], in serum free media (SFM). Panc-1 cells were subjected to serum free medium- SFM; SFM supplemented with ITS; SFM/ITS with Activin-A and SFM/ITS with Swertisin for 8 days. We observed effective cell clustering which started on 3^rd^ day in incubated cells, demonstrating zone of activation for endocrine reprogramming. Activin-A induced clusters turned into mature ILCCs by 8^th^ day and showed presence of insulin, demonstrated by Dithizone (DTZ) staining on 10^th^ day (Fig 1A). Immunostaining for insulin, c-peptide and glucagon showed mature islet formation (Fig 1A). Similar to Activin-A, Swertisin also exhibited intense crimson red to brown DTZ staining whereas those from SFM with ITS failed to stain (Fig 1B). Clusters from all three groups, when examined immunocytochemically, confirmed the presence of deep insulin staining (brick-red) in Activin-A and Swertisin clusters but weakly positive in SFM alone group (Fig 1B).

We further quantitated insulin fluorescence signal per unit cytoplasm, where we found significant increase in insulin signals both in Activin-A and Swertisin clusters upon 8 days differentiation compared to SFM control. Moreover Swertisin group showed even significant high insulin content than Activin-A group (Fig 1C and 1D). Scatter plot analysis between intensity of insulin fluorescence (alexa 488-red) and nuclear signal of DAPI (405-Blue), depicted frequency for insulin immunopositivity in differentiated clusters. When observed in SFM with ITS, scatter showed low distribution towards alexa 488 signals but more nuclear signal pixels, depicting weaker progression for beta cell differentiation fate. Comparatively, a dramatic shift in alexa 488 signals was observed with both Activin-A and Swertisin mediated clusters (Fig 1C), indicated accelerated fate.

### Swertisin follows Activin-A mediated pathway for islet differentiation

In order to understand the mechanism of action of Swertisin for islet differentiation, we carried immunoblotting of key transcription factors in endocrine reprogramming from Swertisin induced clusters at day 10^th^. Cited literature clearly states that Activin-A carried new islet formation via ACT-MEPK-TKK pathway [17–19]. As this mechanism is very well reported, so we tested Swertisin and observed for involvement of MEPK-TKK pathway.

**Fig 2:**
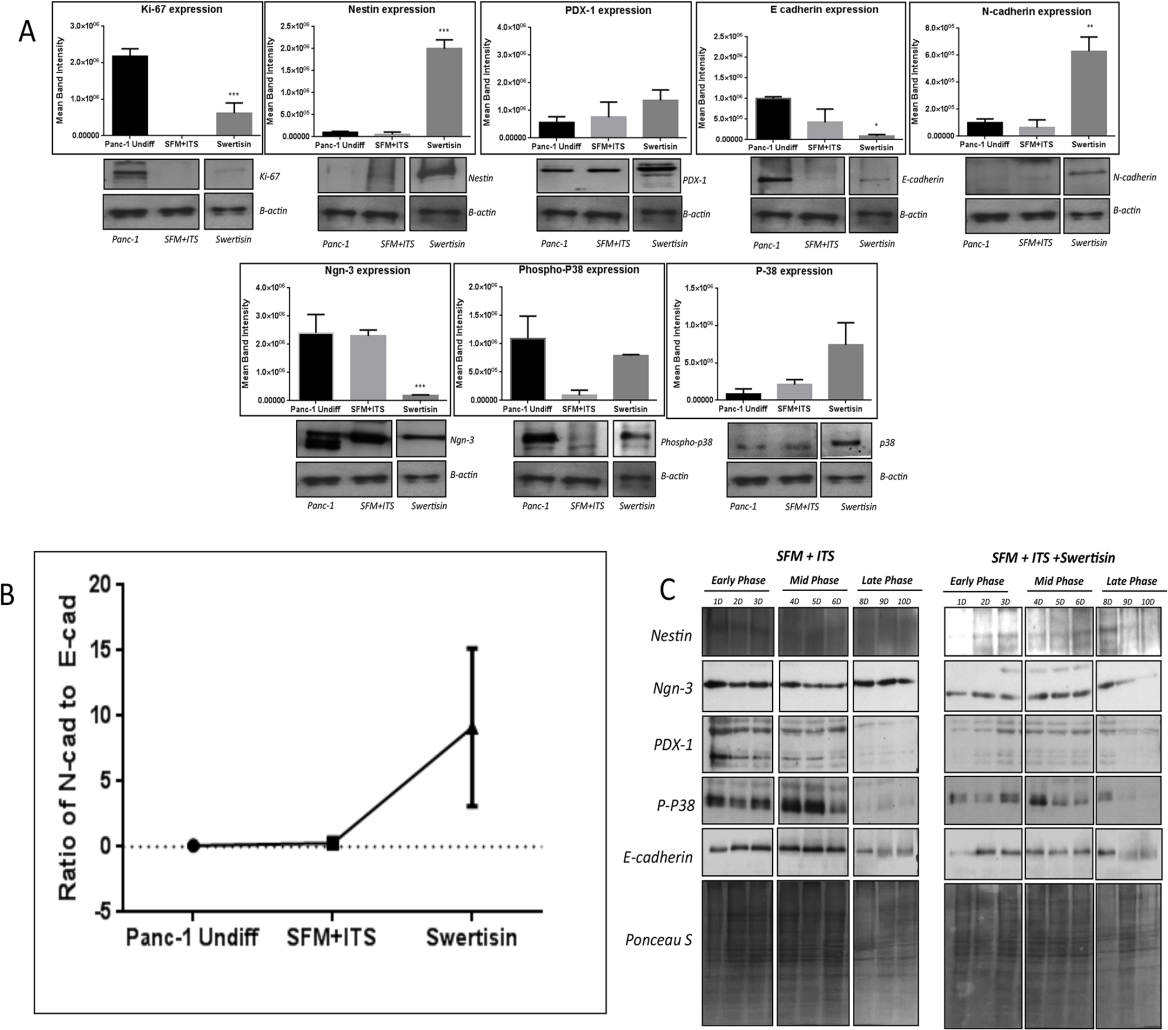
Time course protein profiling and Fate of islet differentiation of Swertisin induced islet differentiation pathway from PANC-1 cells. (A) represents immunoblotting of key parameters that indicate the movement of PANC-1 cells from precursor cells to endocrine islet-like cells, such as stem cell marker Nestin, pancreatic endocrine islet markers Ngn-3 and PDX-1. The activation of p38 MAP kinase to form phospho-P38 and replication marker Ki-67. Protein expression was quantified densitometrically from three independent experiment and expressed as Mean±SEM. *** shows p value <0.001 Vs Panc-1 undifferentiated cells and SFM/ITS. Immunoblotting of E cadherin and N Cadherin demonstrating fate of islet differentiation from undifferentiated PANC-1 cells to endocrine islet-like cells. Graph represents ratio of N-cadherin to E-cadherin depicting transition from precursor sate to differentiated state. Protein expression was quantified densitometrically from three independent experiments and expressed as Mean±SEM. (B) demonstrate characterization of Swertisin induced formation of islet-like clusters from PANC-1 cells in ten days time course. In this experiment shown, cells were harvested each day till ten days and immunoblotted for key parameters that indicate the movement of PANC-1 cells from precursor cells to endocrine islet-like cells in time dependent manner. Cells from control SFM/ITS and Swertisin, harvested from day 1 to 10 were probed for nestin, Ngn-3 PDX-1, phospho-P38 and E-cadherin. (C) shows short-term time course for key proteins implicated in islet differentiation pathway from Swertisin induced clusters. Immunoblotting of nestin, phospo-P38 and Ngn-3 in short-time course manner at 1, 3, 6, and 9 hours induction was performed. Lower images represent graphical representation of movement of protein expression in each group during 1 to 9 hours.

PANC-1 cells in undifferentiated state show high proliferation by persistent ki67 expression and low nestin, PDX-1 and Ngn-3 expression. These cells were found to express high phospho-p38 and depleted basal p-38 (Fig 2A). The presence of high e-cadherin but less n-cadherin expression postulated progenitor nature and undifferentiated state (Fig 2A). Protein lysates of 10^th^ day differentiated islet like clusters, when probed for various transcription factors and MAP kinase signal proteins, revealed substantial inhibitory effect on proliferation rate with low Ki67 expression in both the groups, *i*.*e*. SFM/ITS, and Swertisin, demonstrating differentiation. However, an elevated expression of nestin in Swertisin clusters but not in other group, confirmed strong endocrine reprogramming with Swertisin. Ngn-3 protein at day 10^th^ was found to be significantly down-regulated in Swertisin induced clusters but remained unchanged in SFM/ITS and undifferentiated cells. Low Ngn-3 protein level indicated that PANC-1 cells with Swertisin are enforced to acquire accelerated endocrine phenotype, so the expression decreases significantly as evident in mature islet cells (Fig 2A).

Further, on 10^th^ day, Swertisin clusters showed high p-38 phosphorylation with basal p-38 compared to SFM/ITS but not with undifferentiated PANC-1. Increased phosphorylation indicates accelerated fate of islet differentiation following p-38 mediated activation, which synchronize with ngn-3 upregulation leading to new functional islet cells (Fig 2A). Comparative statistical protein expression reveal that Swertisin at 10^th^ day able to significantly increase high nestin, pre-requisite for differentiation signals, followed by modest increase in PDX-1 expression, with persistent p-38 phosphorylation and significant Ngn-3 down regulation compared to SFM/ITS, conferring beta cell neogenesis (Fig 2A). This can also be supported by ratio of n-cadherin to e-cadherin, which is critical in order to understand the fate of conversion of progenitor from undifferentiated phenotype to differentiated ones. Swertisin clusters after 10 days showed significantly high n-cadherin to e-cadherin ratio, which depicted, differentiated nature of cells whereas SFM/ITS did not show any change compared to undifferentiated cells (Fig 2B).

### Swertisin mode of action involves p-38 phosphorylation and Ngn-3 activation in time dependent manner

Ten-days proteomic analysis highlighted involvement of p-38 but at this time, ngn-3 up regulation was not observed in response to p-38 phosphorylation. Hence, to investigate the expression pattern of ngn-3 in response to p-38 phosphorylation, we did 1–10 days time dependent study for p-38 phosphorylation along with key transcription factors. Immunoblot study from day 1 to day 10 in clusters differentiated with SFM/ITS and Swertisin demonstrated that Swertisin follows differentiation process similar to Activin-A mediated pathway. Samples harvested on each day probed for nestin, ngn-3, pdx-1, phospho and basal-p38 and E-Cadherin (Fig 2C).

We noted that Nestin, a marker for pancreatic progenitor cells in SFM/ITS clusters appears just for initial period, 2–3 days after which it disappears leaving cells undifferentiated, whereas with Swertisin it shoots up by day 2 and remains till day 9, indicating that nestin enforcing these cells to change from precursor type to differentiated cells. The pancreatic duodenal homeobox gene 1 (PDX-1), a marker of pancreatic endodermal cells remained unchanged up to six days with a modest increase during 7–10 days and same is the case with Swertisin as well. Further, the master regulator gene Ngn-3 expression was strongly upregulated in SFM/ITS from day-2 and persists till 10^th^ day whereas in Swertisin, it elevates right away in early phase day-1 and peaks at mid phase day 4–6 with a declined in latent phase day 9 (Fig 2C), following a typical expression pattern during pancreatic morphogenesis. In parallel, since the effect of Activin-A on Ngn-3 expression is mediated via p38 MAP kinase (Figure A and B in S4 Fig), we also noted that phospho-p38 MAP kinase signal increased by day-2 and remains high till day-6 in SFM/ITS, after which it declines. Similar trend was also observed with Swertisin where phospho-p38 MAP kinase signal increases by day-2, peaks at day-4 leading to activation of Ngn-3 in consequence and then declined by day 8, (Fig 2C). The expression of e-cadherin remain unchanged in SFM/ITS cells till last time point while in Swertisin clusters, e-cadherin started to decline by day 6 and went significantly low till day 10^th^ (Fig 2C).

### MAP kinase pathway inhibition using specific p38 inhibitor, SB203580 abrogated Swertisin mediated mode of action for islet neogenesis

**Fig 3:**
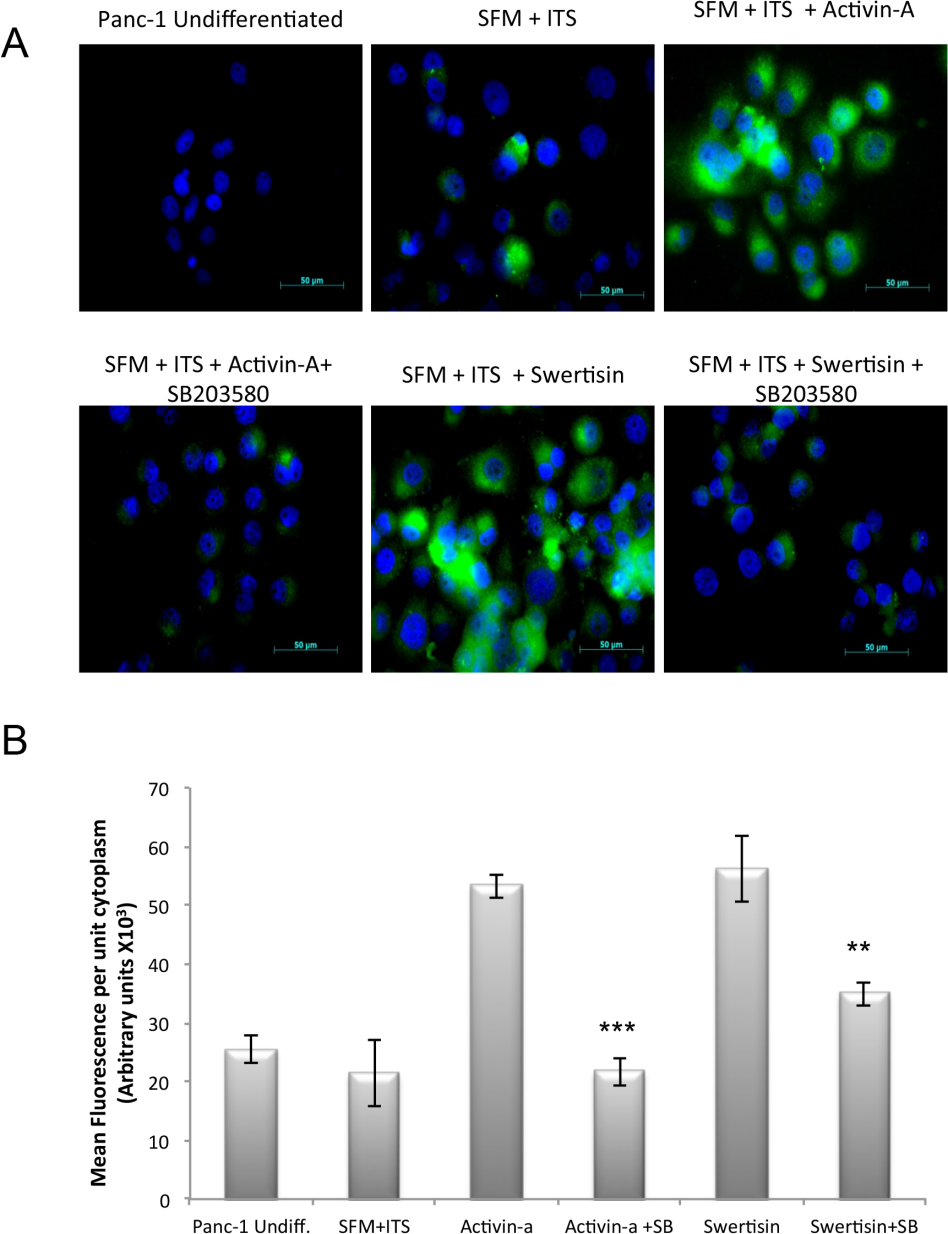
Insulin fluorescence and Insulin content per unit cytoplasm quantification in Swertisin induced islet-like clusters derived from PANC-1 cells with activin-A and MAPKinase Inhibitor. (A) shows fluorescent images for insulin expression in islet differentiation pathway inhibited using p38 MAP kinase inhibitor SB-203580 added in conjunction with Swertisin throughout ten days. The islet-like clusters were immunostained for insulin (green) on 10^th^ day. Nuclear DNA was stained with DAPI (blue). (B) represents insulin content in islet like clusters after p-30 MAPK pathway inhibition. Graph represents insulin fluorescence quantification per unit cytoplasm in differentiated cells with and without inhibition of MAPK pathway using p38 MAP kinase inhibitor SB-203580 added in conjunction with Swertisin throughout ten days. Data is represented as Mean±SEM. *** and ** shows p value <0.001 and 0.01 Vs Activin-A and Swertisin alone groups respectively.

As proteomic studies done above demonstrated pivotal role of p-38 MAP kinase in islet neogenesis, we confirmed its role using strong inhibitor of MAP kinase pathway. We observed that Panc-1 cells when differentiated with either Activin-A or Swertisin, showed deep cytoplasmic insulin immunofluorescence staining (green). Undifferentiated Panc-1 cells failed to stain for insulin, while those of SFM/ITS showed little insulin positive stained cells. In order to confirm, that Swertisin does mediate islet differentiation *via* MAP kinase pathway, similar to Activin-A, we allowed cells to differentiate in presence and absence of specific p-38 MAP kinase inhibitor SB203580 along with Activin-A and Swertisin. We observed significant reduction in insulin positive cells when Activin-A clusters were differentiated in presence of SB203580 (Fig 3A). Similarly, Swertisin induced clusters also fail to synthesis insulin in presence of SB203580 (Fig 3A). Quantitative measurement of insulin fluorescence per unit cytoplasm clearly demonstrated significant retardation in insulin expression in both Activin-A and Swertisin mediated clusters with SB203580 confirming the involvement of MEPK-TKK pathway in islet differentiation, which gets abrogated upon inhibition (Fig 3B).

### Isolated primary cultured mouse intra-islet pancreatic progenitor cells showed islet differentiation with Swertisin induction

Demonstrating the mechanism of action for new islet formation from small molecules like Swertisin using cell line model system PANC-1, enlighten involvement of MEPK-TKK proteins. It is more relevant to reconfirm these facts in primary cultured cell model for pancreatic progenitor cell system. Hence, we isolated mouse intra-islet pancreatic progenitor cells and developed a primary culture. We successfully established a stable cell system which showed initial populate of fibroblast to epithelial morphology by passage-3, but these cells later stabilized and formed pure colonies by passage-5 and complete homogeneous cell population by passage-7-11 (Figure A in S1 Fig). We further characterized these cells for their progenitor nature and found them positive for nestin, vimentin, ngn-3 and pdx-1 expression. Importantly we could show that these cells were negative for insulin staining at this time, which confirms their undifferentiated state (Figure A in S1 Fig).

We further confirmed the presence of progenitor marker and monitored protein expression using immunoblotting in these cells at different passages right from isolation till it develops into stable cell line. Nestin a marker of pancreatic progenitor, was very weekly expressed at passage-5, increased gradually by passage-7, 9 and highly peaked at passage 11 and remained constant thereafter. E-cadherin, expressed by stem/progenitor nature cells, was also found to be increase in gradual manner from passage 7 till 11. Similarly, Vimentin showed increased expression pattern by passage7-11. More likely, Basal expression of pdx-1 and ngn-3 was found right from passage 5 till passage 9, which signifies that cells are maintaining their progenitor state. Extremely low expression of n-cadherin depicted undifferentiated state of these cells in all passages (Figure B in S1 Fig).

### Swertisin acts via p-38 MAP kinase and involves Smad2/3 transcriptional regulation

**Fig 4:**
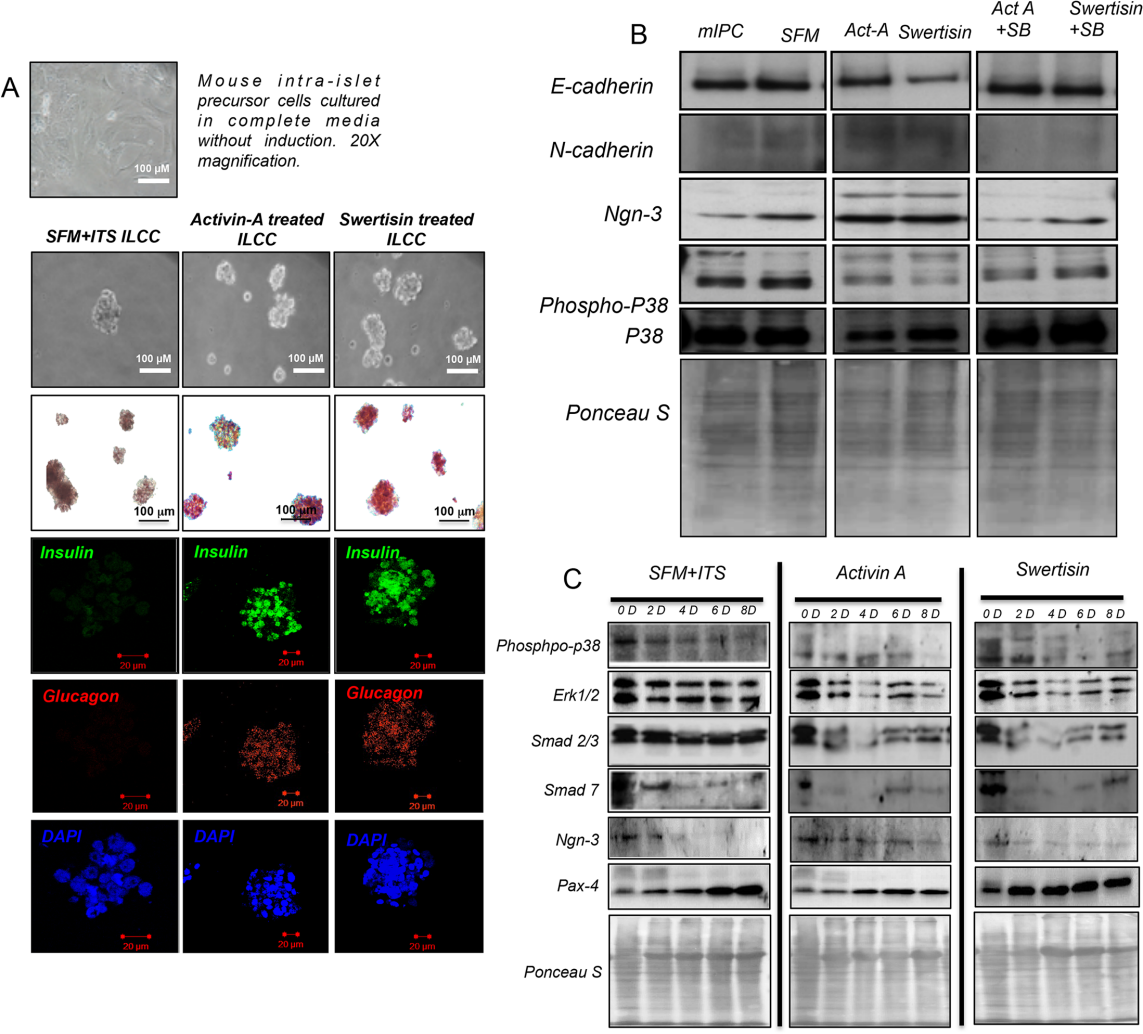
Differentiation of mouse intra-islet progenitor cells and immunoblotting of key transcription factors and islet markers under differentiation with activin, swertisin and presence of p-38 MAPK inhibitor. (A) depicts mIP cells cultured in complete media at day 0 which were then subjected to differentiation using activin-A and swertisin for 10 days. Bright field image shows, cells under differentiation on 8^th^ day at 20X magnification, and dithizone stained clusters on day 10^th^. A fluorescent image represents immunostaining for insulin (green) and glucagon (red) in clusters from SFM/ITS, activin-a and swertisin groups. DAPI was used as nuclear stain. (B) shows immunoblotting of E-cadherin, N-cadherin, Ngn-3, P-p38, Native p-38 MAP kinase pathway proteins in presence and absence of MAP kinase pathway inhibitor SB203580. Ponceau S stain blot was shown as loading control. (C) shows immunoblotting of key differentiation pathway parameters that indicate the conversion of Mouse intra-islet progenitor cells into islet like clusters. Key transcription factors and MAP Kinase pathway proteins like P-p38, Erk1/2 Ngn-3, Pax-4, and Smad proteins under differentiation were monitored. Beta actin was used as loading control.

Mouse intra-islet progenitor cells were subjected to islet differentiation with SFM/ITS, Activin-A and Swertisin. Islets like clusters were formed with both Activin-A and Swertisin in 8-day protocol. Clusters thus formed were found positive for deep crimson red DTZ staining showed presence of insulin. The islets generated from each group were immunostained for insulin and glucagon. SFM/ITS did not show evident insulin (green) and glucagon (red) staining, while both Activin-A and Swertisin clusters showed intense insulin and glucagon staining (Fig 4A).

Further, we observed immunoblotting profile of key proteins involved in islet differentiation pathway on 10^th^ day and reconfirmed the role of p38 MAPK using its inhibitor SB203580. Undifferentiated mIPC and SFM/ITS clusters showed high e-cadherin but no n-cadherin. Also ngn-3 was not activated in spite of high p38 phosphorylation (Fig 4B). On other side Activin-A and Swertisin differentiated clusters showed presence of n-cadherin followed by elevated ngn-3 expression triggered by p38 phosphorylation which goes depleted by this time. More interestingly, when clusters differentiated with SB203580 were probed, we found that n-cadherin was lost by this time and ngn-3 failed to upregulate with low p-38 phosphorylation (Fig 4B) these observations again highlighted that Swertisin mediate islet differentiation involving p38 MAPK through MEPK-TKK pathway

Further we attempted to monitor downstream signaling of p-38, involving Smad protein complex 2/3 and Smad 7 playing pivotal role in differentiation signaling and control of transcription factor expression like ngn-3. We compared SFM/ITS, Activin-A and Swertisin together for Smad2/3, Smad-7 in response to phospho-p38 in time dependent manner from day 0 till day 8. SFM/ITS clusters showed high Smad 2/3 and Smad 7 expression which remain elevated till day 8, whereas Activin-A and Swertisin both molecules showed initial Smad2/3 levels, which goes down progressively over differentiation process and maturation along with steep decline in, Smad-7 right from day-2 with p-38 phosphorylation (Fig 4C). In continuation, we also observed levels of Erk1/2 along with p-38, where SFM/ITS group showed high basal Erk1/2, which remains continued till day 8. However both activin and Swertisin showed gradual depletion in erk1/2 levels right from the beginning, indicating progression of differentiation by possibly phosphorylation of erk1/2 in response to phospho-p38. Further, we also observed pax-4 expression for maturation of progenitors into beta cells lineage. SFM/ITS showed a very delayed pax-4 expression at day 6–8. However activin-A could show pax-4 increase by day4, while Swertisin triggers pax-4 increase right from day2 till day 8 (Fig 4C). These evidences confirm that p-38 proceed with Smad protein complex recruitment as reported with Activin-A mediated TGF-beta signal transduction during pancreatic morphogenesis [8].

### MAP Kinase along with Smad proteins signals facilitate endogenous islet differentiation by Swertisin in-vivo

Ppx has been reported to involve beta cells replenishment by islet neogenesis from stem/precursor cells. In present experiment we made an attempt to investigate the mechanistic action of Swertisin induction for new islet formation mediated through MEPK-TKK pathway, under *in-vivo* condition in surgically induced pancreatectomised mice.

We observed that mice undergone 70% Ppx with and without Swertisin did not demonstrate any change in body weight during three days post PPx, compared to sham operated control mice (Data not shown). An increasing trend in remnant pancreas tissue weight has been observed in Swertisin treated animals on 3^rd^ day, notifying regeneration of small fraction of new pancreas, although it is not statistically significant compared to control animals (Figure B in S2 Fig). Fasting blood glucose also remained unchanged in both the groups over three days (Figure C in S2 Fig).

In order to confirm the islet differentiation, mRNA expression for various islet specific transcription factors was observed using RT-PCR. Semi quantitative gene expression data revealed that Ppx alone animals favored activation of pancreatic progenitor gene–PDX-1 and doesn’t reach to endocrine fate commitment until 3^rd^ day post Ppx. On the other hand, Swertisin treated Ppx mice identified low PDX-1 but more Ngn-3 expression suggesting early induction and endocrine fate regeneration signals (Figure A and B in S3 Fig). None of the animal group had shown nestin expression, but Swertisin treated PPx mice reveled significantly elevated insulin gene mRNA transcripts compared to Ppx alone (Figure B in S3 Fig).

**Fig 5:**
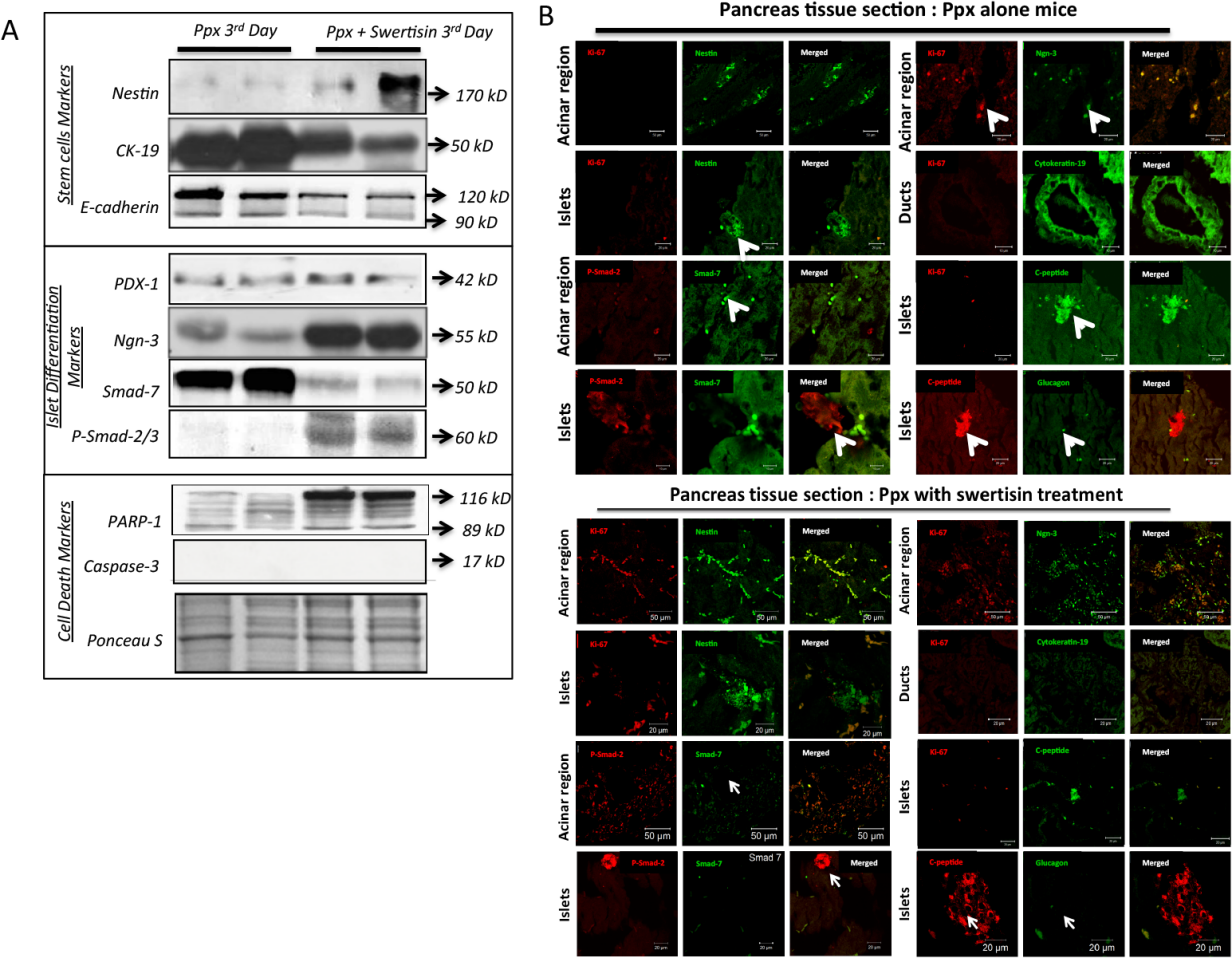
In-vivo analysis of molecular mechanism by Swertisin differentiation in Ppx mice model. (A) confocal images from regenerating pancreas for assessment of various islet transcription factors and signaling proteins of TGF-beta pathway from tissue of Ppx sham and Swertisin treated animals. Various markers like Ki67, Nestin, Ngn-3, CK19, p-smad-2, Smad-7, Insulin and Ck-19 were probed and analyzed. (B) showed western blot profile of stem cell markers, key transcription factors in islet differentiation pathway and cell death markers in PPx Sham and Swertisin treated animal pancreatic tissues.

More importantly, protein lysates extracted from regenerated pancreatic tissues on 3rd day were probed for islet, MAP Kinase and Smad transcriptional pathway proteins. In western blot analysis we found that Ppx alone animals fails to induce nestin expression as noted in mRNA data earlier, whereas Swertisin treated Ppx animals persist some nestin expression leading to early differentiation signals (Fig 5A). Further the marker of ductal progenitor cells Cytokeratin-19 (CK-19) was highly upregulated in Ppx alone mice depicting ductal fibrosis (duct cell proliferation) which was significantly down regulated in Swertisin treated animals. E-cadherin which is known to express more in progenitor cells compared to differentiated cells has been observed in high proportion in Ppx alone animals but low expression was recorded in Swertisin treated mice. Interestingly, at protein levels we did not find any significant change in PDX-1 expression in both the groups, but key master regulator ngn-3 expression was significantly upregulated in Swertisin treated mice compared to untreated ones. This dramatic increase in ngn-3 expression in Swertisin treated mice confirmed the stated role of p38 MAPK as a trigger for MAPK-TKK pathway and neuro-endocrine differentiation signals. To further confirm this, the downstream TF like p-Smad2, 3 and 7 were analyzed. When compared, we found Ppx alone mice expressed significantly high Smad 7, whereas samd-2 phosphorylation, which is a prerequisite for induction of islet differentiation pathway, was high in Swertisin treated animals (Fig 5A).

### Activation of MAP Kinase leading to Smad regulatory mechanism is responsible for new islet cells mass in regenerated pancreas

We then observed and confirmed p-38 mediated activation of ngn-3 expression leading to islet neogenesis in regenerated excised pancreatic tissues from Ppx alone and Swertisin treated animals (immunohistochemistry) by probing various markers for islet differentiation pathway and Activin-A signaling pathway. In Ppx tissues we found that nestin expression (green) was randomized in some patches near acinar tissue, but few islet cells showed nestin positivity as well. None of these areas showed ki67 expression (red) indicating less proliferation of acinar and islet cells. A persistent proliferating population of ngn-3 positive (green) cells was observed with dual ki67 staining (red). Moreover Ppx animals also showed intense Ck19 (green) staining in epitheloid ductular structures. Most important observation was that PPx animal tissue had deep smad-7 (green) staining and very weak p-Smad 2 (red) staining. The presence of low c-peptide with few scattered glucagon positive cells and compact size islet were observed indicating slower differentiation process in newly regenerated untreated (Sham) mouse pancreas (Fig 5B). On the other hand, Swertisin treated animal tissue showed large number of dual stained population of ki-67 and nestin within both acinar and islet tissues. These sections also showed low Ck19 staining in ductular tissues, but enormously high p-smad-2 staining within acinar and islet area. The staining of smad-7 was lower in treated animals suggesting presence of few progenitor nature cells with faster differentiation (Fig 5B).

## Discussion

In the present study, we have reported that Swertisin treatment promoted the differentiation of human and mouse pancreatic progenitor cells mediated through MAP kinase pathway (MEPK-TKK pathway) similar as of Activin-A and could be inhibited for terminal differentiation into endocrine cells in the regenerating pancreas if treated with MAP kinase specific inhibitor SB203580.

Pancreas (organ transplant) or Cadaveric islet transplantation treatment strategies have many limitations [20]. Stem cell to islet differentiation provides alternative approach to cure both Type-1 and Type-2 disease [21]. Currently efforts are being made to generate insulin-producing cells from stem cells or tissue specific progenitor cells [22, 23]. Very few reports are there which show pancreatic regeneration using herbal extract or compounds. One of such study done by Kojima et al. showed insulin positive cell differentiation from AR42J cells using conophylline isolated from Ervatamia microphylla [24].

*Enicostemma littorale* is one such anti-diabetic plant, which we have examined for its capacity of promoting islet neogenesis reported earlier [14, 15]. *In-vitro* formation of islet-like cell clusters containing both β and α cells were observed, when treated with herbal bioactive agent Swertisin isolated from *E*. *littorale* [15]. This led us to investigate the molecular mechanism of this potent bioactive compound “Swertisin” for its effective islet cell differentiation property. We hypothesized that Swertisin might differentiate progenitor cells undergoing pathway same as that of Activin-A via MEPK-TKK pathway. To test the mode of action of Swertisin, we used human pancreatic progenitor panc-1 cells and mouse intra-islet primary cultured pancreatic progenitor cells. We compared the crucial transcription factor expression playing inevitable role in islet neogenesis with the clusters generated from both Swertisin with Activin-A in time dependent manner. Exogenous Activin-A treatment increases the proportion of insulin cells in the developing chick pancreas [25]. Activin A also induces differentiation of human fetal pancreatic endocrine cells [26]. Previously few groups have shown that panc-1 cells and primary cultured islet progenitor cells could differentiate into islet like clusters using Activin-A [27, 28], so it become evident to investigate the mechanism of action in new islet generation targeting Activin-A mediated TGF-beta pathway.

In our experiment, Swertisin effectively differentiates panc-1 cells and mIP cells like Activin-A. Further we demonstrated that within ten days of treatment with Activin-A and Swertisin, islet-like clusters were formed that could stain with DTZ and immunostain for insulin and glucagon both. Comparative quantification of insulin content per unit cytoplasm during differentiation in clusters postulated the fact that Swertisin demonstrated, even more efficient in differentiating capacity compared to Activin-A. Cells under differentiation stopped proliferation and showed significant decline in Ki-67 expression According to the literature, islet differentiation pathway starts with either nestin progenitor cells [29, 30] or by activation of master key gene ngn-3 [31, 32]. Nestin was expressed at different levels in the acinar component, as well as in ductal structures and islets to some degree. We have also noted that Nestin, a key marker in islet differentiation signaling, peaks at early time and subsequently declined at later time in differentiation, with strong up regulation of Ngn-3 between mid times in Swertisin mediated clusters from panc-1 and mIP cells. Number of investigators used pdx-1 as candidate master regulator in islet generation where pdx1 protein directly binds and activates the insulin promoter [33], and at least one acinar enzyme gene [34]. Further, Baeyens et al., in 2006 demonstrated that *in-vitro* growth factor stimulation could induce recapitulation of an embryonic endocrine differentiation pathway in adult dedifferentiated exocrine cells via ngn-3 ectopic expression and over activation [32]. In our study we found a very steady expression of pdx-1 in differentiated clusters from start to end, demonstrating no significant impact of pdx-1 over differentiation process. Moreover ngn-3 was the key factor, which showed high upregulation both in cell line and primary culture system. Swertisin, showed high ngn-3 cells in response to p-38 phosphorylation, which highlighted the pivotal role of p-38 MAP Kinases in this process.

Earlier reports demonstrated that Activin-A a member of TGF-β family of proteins that helps in growth and differentiation of β-cells [8, 26], promotes expression of pro-endocrine gene ngn-3 by upregulating p38 MAP kinase [24] through Smad 2/3 phosphorylation and Smad 7 down regulation [8]. In the present study the signaling pathway of both Activin-A and Swertisin is found to be mediated through pro-endocrine gene ngn 3 triggered by of p38 MAP kinase phosphorylation [24], we then confirmed whether SB203580, an inhibitor of p38 MAPK could abolish islet-promoting action. With inhibition of MAP kinase signaling, Activin-A failed to form islets, and the same was observed with Swertisin in both human and mouse cell systems. These facts confirmed that Swertisin do form islets mediated by ACT-MEPK-TKK pathway. Further when looked downstream to p-38, Smad protein showed high Smad2/3 phosphorylation and reduced Smad 7 expression *in-vitro* and *in-vivo* experiment both with Activin—A and Swertisin.

Ppx is well known model for the study of β-cell regeneration. The mechanism by which regeneration occurs in this model has been controversial, with some claiming that islet and β-cell neogenesis is important, while others claim that β-cell replication is predominant [35, 36]. The process and mode of regeneration efficiency that follows with Ppx injury depends on the degree of surgical insult. In 30–50% Ppx, β-cell mass expansion is mainly due to replication of pre-existing β-cells, but no ductal cell proliferation or Ngn-3 induction. However with 70–90% Ppx, the β-cell mass expansion has been attributed to neogenesis with the proliferation of putative ductal precursors in main pancreatic ducts, and induction of Ngn3 expression. Also there is a wave of proliferation initiated in main duct, which is followed in smaller ducts and finally in islet β-cells, with transient up-regulation of PDX-1 in ductal cells along with nestin and ngn-3 [37]. Also it is interesting to note from literature that activin is upregulated in duct cells following partial pancreatectomy and streptozotocin injection, suggesting that activins might be involved in the initiation of β-cell neogenesis following distinct stimuli in adulthood [38, 39]. On this basis we again examined and reconfirmed the mechanistic action of Swertisin in *in-vivo* condition with 70% Ppx mice model. Seventy percent Ppx animals were treated with single injection of Swertisin in pancreas on day 0, showed high ngn-3 expression by 3rd day with elevated p-samd2 and low samd-7, indicating ACT-MAPK pathway activation. Moreover less e-cadherin and ck-19 expression again reconfirms low ductal cell proliferation, opposite of which was observed in sham operated control animals. Altogether, our *in-vivo* data does present evidences in accordance with earlier *in-vitro* observation made by us [14, 15].

**Fig 6:**
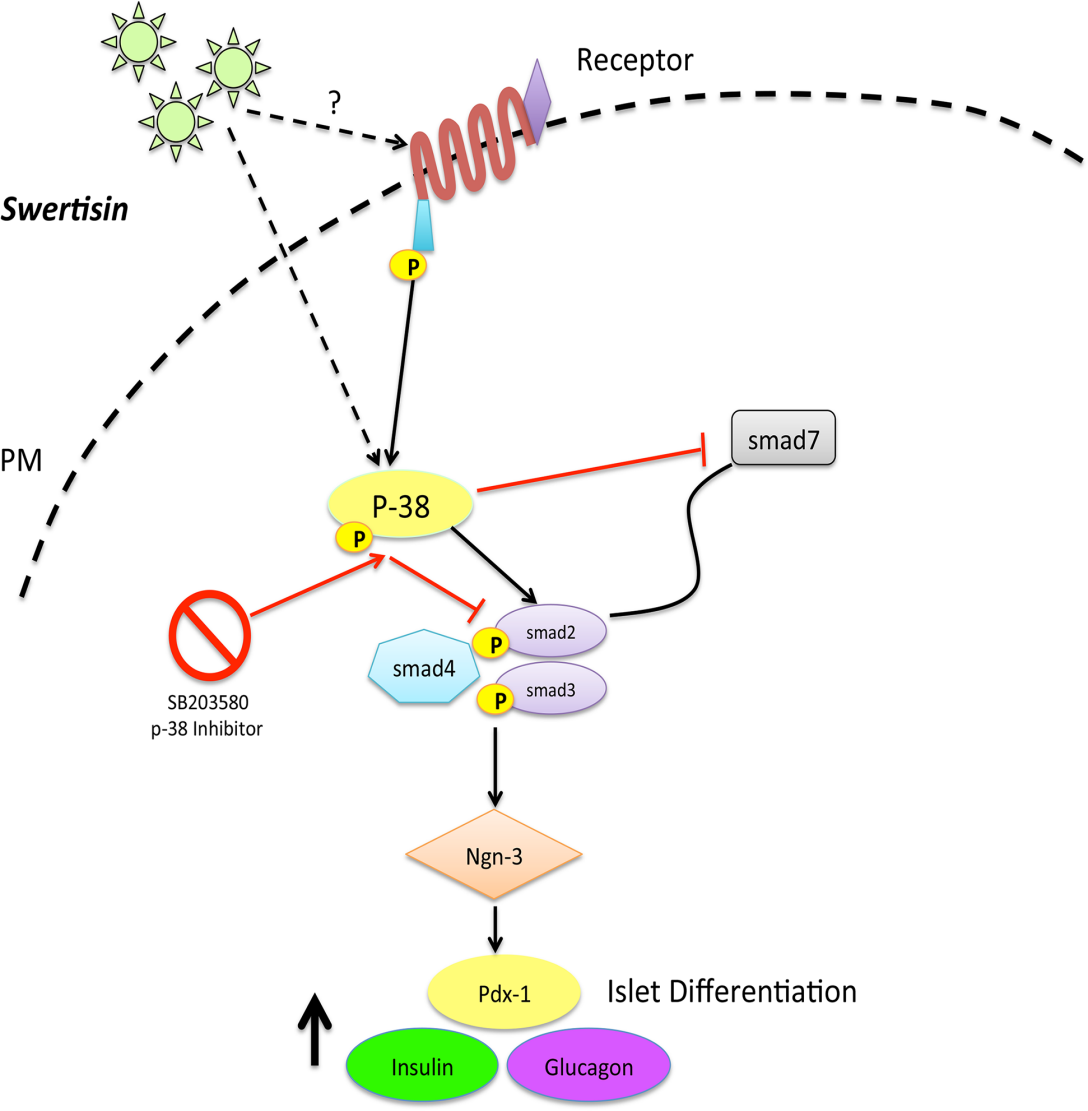
Proposed Mechanism of action of Swertisin mediated Islet Differentiation. Panel shows proposed mechanistic action pathway for islet neogenesis being followed by Swertisin for new islet cell differentiation.

We therefore collectively postulate an underlying mechanism of action for Swertisin mediated by nestin overexpression triggering MAP kinase pathway that involve p-38 MAP kinases and Smad proteins. These phosphorylated proteins recruiting a complex of Smad protein (2/3 and 4) induce differentiation while Smad-7 maintaining progenitor state in these cells. Smad complex then go and bind to DNA for regulating and activation of important key regulatory transcription factors such as ngn-3 (could be one target) and facilitate endocrine reprograming under MAP kinase signals (Fig 6). Elevated neurogenin-3 expression work as a driving force for these cells to undergo endocrine reprograming and form new islet like cell clusters via p-38 MAPK signals which gets abrogated by p-38MAPK inhibitor, when added (Fig 6).

## Conclusion

In the present study, we first time reported the mechanistic action of a potential natural islet neogenic agent Swertisin and demonstrated this islet forming action by tracing expression of crucial transcription factors and MAP kinase pathway with and without inhibition using human pancreatic progenitor panc-1 cells, mouse intra-islet progenitor and 70% Ppx mouse models. Swertisin differentiate stem/progenitor cells into insulin producing cells via MEPK-TKK pathways followed by TGF-β (Activin-A mediated) pathway for new islet clusters formation both *in-vitro* and *in-vivo* via nestin driven p-38 phosphorylation in-turn regulating ngn-3 overexpression. Moreover, the information of endogenous islet neogenic potential seems to be very useful in designing newer therapeutic approaches and target molecules, governing TGF-β mediated signaling pathway to treat diabetes mellitus.

## Material and Methods

### Cell culture maintenance

Human Panc-1 cells, as described earlier by Lieber M et al. [40], were obtained from Dr. Girish M Shah’s Lab. Universite Laval, Quebec, Canada (as generous gift) and maintained in high glucose DMEM supplemented with 10% Fetal Bovine Serum. Cultures were maintained in 95% air/5% CO2 at 37°C, and the medium was replenished every alternate day. Cells were regularly monitored for subculturing and trypsinized with 0.5% TPVG (Sigma Aldrich, USA) at 80% confluency.

### Isolation and establishment of mouse intra-islet progenitor cells

Mouse intra-islet progenitor cells were isolated from fresh islet preparation followed by digestion of whole mouse pancreas. Mouse pancreata were dissected and subjected to collagenase digestion (Sigma Aldrich, USA), as described previously [41]. Two pancreata were used per isolation, and islet isolations were performed for each time point in the study. Purification was achieved and the final preparation was confirmed with 90% dithizone-positive structures. Freshly isolated islets (5000 islets per 25 cm2 flask) (Corning, Fisher Scientific, Canada) were placed and cultured in RPMI-1640 complete media with 10% serum (Gibco, ON, Canada) for 24 hours and then shifted to DMEM complete media with 10% serum (Gibco, ON, Canada) to promote monolayer formation. Cultures were maintained in 95% air/5% CO2 at 37°C, and the medium was renewed every alternate days. The monolayers were subcultured when the cells had grown to near confluence. Representative islet cultures were examined immediately after isolation (day 0), and the derived monolayers were examined at 1, 5, 7, 9 and 11 passages of the culture period in the subsequent investigations.

### Differentiation of Panc-1 and mIP cells into islet like clusters

The cells were allowed for differentiation in presence of Serum free medium, Activin-A and Swertisin as previously described [14]. Normal Panc-1 cells and mIP cells were differentiated using Activin-A at 20 ng/ml and Swertisin at 15μg/ml concentrations as differentiating factors in a eight day differentiation protocol with insulin (5μg/ml) transferrin (5μg/ml) and selenite (5ng/ml) cocktail (Sigma, Aldrich USA). In order to better comprehend the differentiation procedure of islet neogenesis with Panc-1 and miP cells, differentiation in presence and absence of SB203580 (Inhibitor of p-P38-MAPK) was performed to understand role of p38 MAP Kinase. In one of the groups Activin-A and Swertisin was added in combination with SB203580, a specific inhibitor of p-38 MAP Kinase at 10μM concentration.

### Immunohistochemistry/Immunocytochemistry

In order to understand the mechanism of Swertisin action in *in-vitro* generated clusters form Activin-A and Swertisin and in regenerated pancreas after 70% pancreatectomy, various islet and key differentiation protein markers were probed immunochemically. Undifferentiated Panc-1 cells and mIP cells were also assessed for their progenitor nature in similar way. Immuno-cyto/histo-chemistry of the following protein markers: Ki-67, Nestin, P-Smad-2, Smad-7, C-peptide, Glucagon, Ck-19, Pdx-1 and Ngn-3 were done. Insulin and Glucagon immunocytochemistry staining was also done to characterize clusters obtained after differentiation from panc-1 cells and mIP cells. Briefly, growing cells or clusters were fixed with 4% PFA for 10 min at 4°C and washed once with PBS, thereafter permeabilized with 0.1% triton X-100 solution in PBS form 5 min at 4°C. Once permeabilization done, samples slides were blocked with blocking solution containing 0.5% BSA with 4% FBS in PBS for 1 hour at room temperature. Slides were then probed with respective primary antibody at desired dilution as shown in S1 Table. Followed with 3 time washes in wash buffer (one tenth of blocking buffer), slides were labeled with fluorescent dye conjugated secondary antibody (dilution shown in S1 Table). Nuclear counterstaining was done with DAPI at 300nM and finally mounted with vectashield mountant for imaging on fluorescent microscope. Images were captured with Nickon TE200S inverted fluorescent microscope and data was analyzed with NIS element Advance version software (Nickon, Japan).

### Protein extraction and Western blotting

Protein samples from various cells differentiated clusters and dissected pancreatic tissue on day-3 post Ppx was harvested and homogenized. Followed with protein isolation, total protein was estimated, which was further analyzed by western blot profiling of the following proteins: Nestin, E-Cadherin, N-cadherin, PDX-1, Neurogenin-3, Phospho-P38, P-38, Smad-7, P-Smad-2/3, Parp-1 and Caspase-3. Cells/ clusters/ tissues were lysed with urea containing lysis buffer (1mM EDTA, 50 mM Tris-HCl pH 7.5, 70 mM NaCl, 1% Triton, 50 mM NaF) supplemented with protease inhibitor cocktail (Fermentas INC.). Protein estimation in all samples was carried out using Bradford reagent according to manufacturer’s suggestions (Biorad, USA). Cell lysates (50μg) were separated on Polyacrylamide gel using Mini-tetrapod electrophoresis system (Biorad, USA) and transferred onto nitrocellulose blotting membrane (Thermo Inc.). Blots were then incubated with blocking milk buffer (5% fat free skimmed milk with 0.1% Tween-20 in PBS). Dilutions of primary antibodies used, against various proteins, listed in S1 Table. Primary antibodies were added to blots and incubated overnight at 4°C. Anti-rabbit and Anti-mouse IgG conjugated with HRP were used to develop the blots using Ultra sensitive enhanced chemiluminiscence reagent (Millipore, USA) and images were captured on Chemigenious gel documentation system (Uvitech, Cambridge).

### RNA extraction, First Stand c-DNA synthesis and semi quantitative Reverse Transcriptase PCR (RT-PCR)

Pancreatic tissue of control and treated mice with Swertisin on the 3rd day post Ppx were dissected and subjected to RNA isolation, first stand c-DNA preparation followed by gene expression profiling by semi quantitative RT-PCR for Nestin, PDX-1, Ngn-3, INS and G6PDH. Total RNA was isolated with TriSoln (Sigma Aldrich, USA) and quantified on Shimadzu Nanospectrophotometer (Shimadzu, Japan) at lambda 260 and 280 nm. RNA integrity and purity was checked on a 1.5% denaturating agarose gel electrophoratically. Followed this 1 mirogram of RNA was reverse transcribed into cDNA using first strand c-DNA synthesis kit (Fermentas INC., USA) as per manufacturing instruction manual. RT-PCR was done with optimal conditions for various genes (see S2 Table) by running gradient PCR performed with a range of annealing temperature from 51–60°C. One μl of cDNA products was used to amplify genes using Fermentas 2X master mix containing 1.5 μl Taq Polymerase, 2mM dNTP, 10X Tris, glycerol reaction Buffer, 25mM MgCl2, and 20pM appropriate forward and reverse primers for each gene (for sequence and details see S2 Table). PCR products were then resolved on a 1.5% Agarose gel (Sigma Aldrich, USA) and visualized with ethidium bromide staining. Images were analyzed using Alpha Imager software (UVP image analysis software systems, USA).

### Animals

Male balb/c mice, 3–4 weeks old, weighing around 25–30 grams, were used for islet isolation for the generation of mIP cells and 70% partial pancreatectomy to understand the mechanism of pancreatic regeneration process *in-vivo* with Swertisin treatment. This study was carried out in strict accordance as per the guidelines of Committee for the Purpose of Control and Supervision on Experiments on Animals, India (CPCSEA). Post experiment animals were euthanatized using xylazine (10 mg/kg) and ketamine (150mg/kg) injection followed by cervical dislocation ensuring death. The protocol was approved by the Committee on the Ethics of Animal Experiments from our institution- The Maharaja Sayajirao University of Baroda, Gujarat, India.

### Partial Pancreatectomy

Seventy percent partial pancreatectomy was performed according to Bouwens et al., and Bonner-weir et al. [22, 29]. Briefly, Mice were anesthetized by administration of ketamine and Xylazine (100 mg/kg and 50 mg/kg bwt; i.p.). Hairs were cleaned and the abdomen was opened through a left lateral incision. The entire splenic portion and most of mesenteric portion of the pancreas was surgically removed, resulting in ~70% pancreatectomy, confirmed by weighing the removed and remnant portions. Sham operation was performed, by opening the abdomen while leaving the pancreas intact. The incision was closed using 4–0 silk treads for the peritoneum and sutured back for the outer skin as well. Animals were given injections of pain reliever for 3 days and wounds were dressed with tropical skin ointment.

## Supporting Information

S1 Fig Isolation and Immuno-characterization of Mouse Intra-islet pancreatic progenitor cells. (Figure A) demonstrate establishment of mouse intra-islet progenitor cells isolated using collagenase type-5 digestion. Purified islets were cultured in condition growth medium and passaged in subsequent generations. Cells at passage no 3, 5 and 7 were captured in bright field image. Immunofluorescence staining shows ngn-3 (TRITC-red), vimentin (cy5-red), nestin (TRITC-red), pdx-1 (FITC-green) and insulin (FITC-green). DAPI was used to counterstain nuclei (blue). (Figure B) shows immunoblotting of key parameters that indicate the establishment of Mouse intra-islet progenitor cells from isolated islet cells in passaging. Key stem/progenitor markers like Nestin, E-cadherin, mesenchymal stem cell marker Vimentin pancreatic endocrine islet markers Ngn-3 and PDX-1, and cell differentiation marker N-cadherin was used. Ponceau S stain blot was shown as loading control.
(TIF)

S2 Fig Swertisin molecule structure, Pancreatic Tissue weight and Fasting glucose in Ppx animals. (Figure A) showing Swertisin molecular structure. (Figure B) graphs demonstrate change in pancreas weight calculated on 3^rd^ day post sacrifice and (Figure C) shows fasting blood glucose in sham operated and Swertisin treated animals.
(TIF)

S3 Fig Gene Expression and histology of Ppx mice tissues. (Figure A-B) shows gene expression data for various islet specific markers in these regenerating pancreatic tissues. Quantitate mRNA expression of genes expressed in post Ppx with and without Swertisin treatment was analyzed. All data seta are represented as mean ± SEM and calculated from 3 independent animal observations. *** and ** represents p value < 0.001 and 0.01 Vs Ppx animals. (Figure C) shows images of pancreas regeneration at 10 day, demonstrating beta cell regeneration
(TIF)

S4 Fig Immunoblot profile of Activin-A mediated islet differentiation pathway. (Figure A) shows western blot profile of activin-A mediated islet differentiation in panc-1 ILCC in time dependent manner. (Figure B) shows protein profile of key differentiation markers in short time 0–9 hours.
(TIF)

S1 Table List of antibodies used in IHC/ICC and immunoblot. Shows list of primary antibodies used in ICC, IHC and western Blot experiments with particular details for each experiment like specificity, dilution factor, molecular weight etc.
(DOCX)

S2 Table List of primer sequences used in RT-PCR. Shows list of forward and reverse primer sequences on all genes used in RT-PCR experiments along with melting point and amplicon size for each gene.
(DOCX)
