# Supplementary figures and images for: Swertisin an Anti-Diabetic Compound Facilitate Islet Neogenesis from Pancreatic Stem/Progenitor Cells via p-38 MAP Kinase-SMAD Pathway: An In-Vitro and In-Vivo Study
Source: PLoS One. 2015 Jun 5;10(6):e0128244. doi: 10.1371/journal.pone.0128244 (PMC4457488; doi:10.1371/journal.pone.0128244)

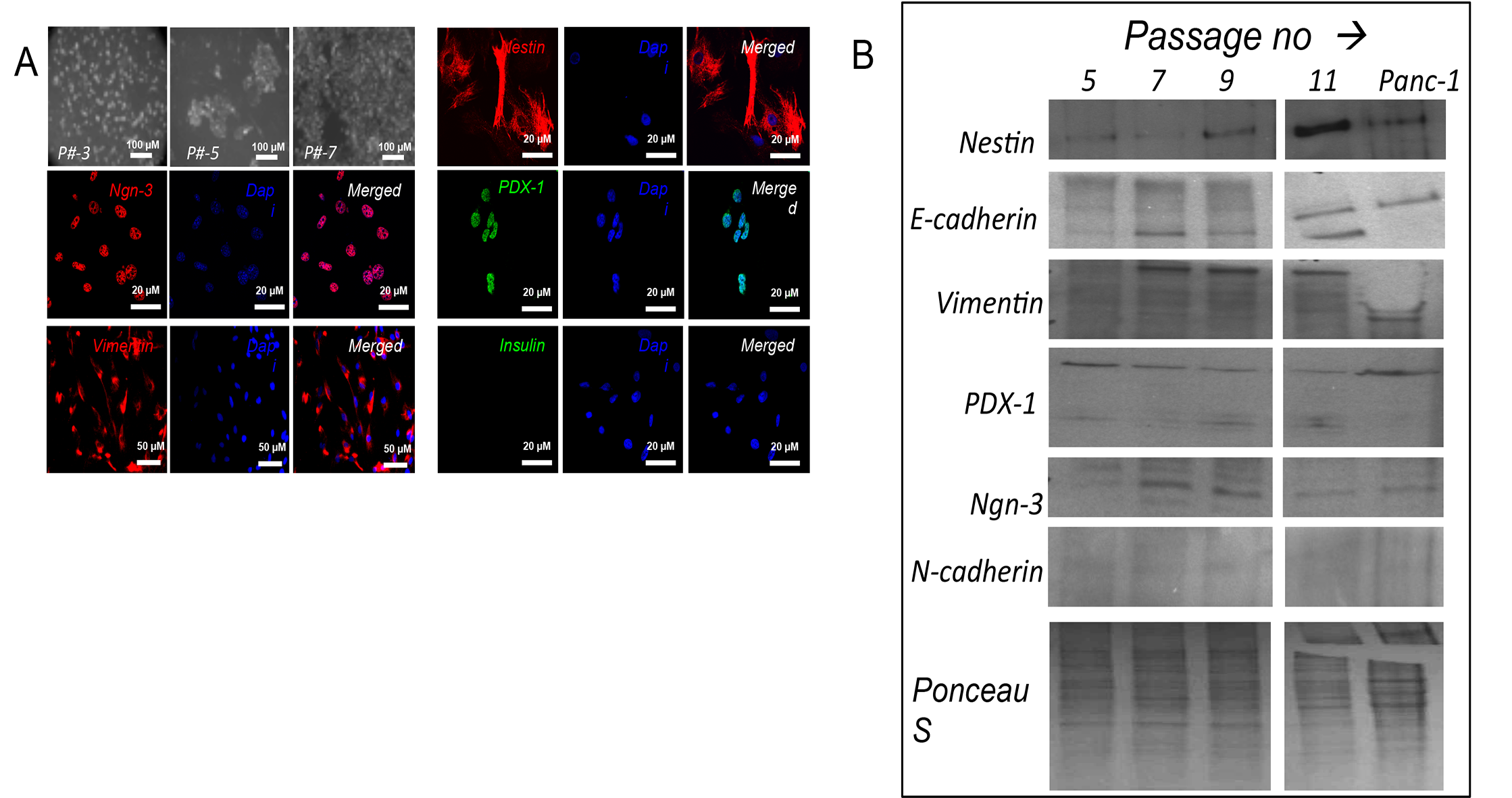

Supplement: S1 Fig — (Figure A) demonstrate establishment of mouse intra-islet progenitor cells isolated using collagenase type-5 digestion. Purified islets were cultured in condition growth medium and passaged in subsequent generations. Cells at passage no 3, 5 and 7 were captured in bright field image. Immunofluorescence staining shows ngn-3 (TRITC-red), vimentin (cy5-red), nestin (TRITC-red), pdx-1 (FITC-green) and insulin (FITC-green). DAPI was used to counterstain nuclei (blue). (Figure B) shows immunoblotting of key parameters that indicate the establishment of Mouse intra-islet progenitor cells from isolated islet cells in passaging. Key stem/progenitor markers like Nestin, E-cadherin, mesenchymal stem cell marker Vimentin pancreatic endocrine islet markers Ngn-3 and PDX-1, and cell differentiation marker N-cadherin was used. Ponceau S stain blot was shown as loading control. (TIF) [file pone.0128244.s001.tif]

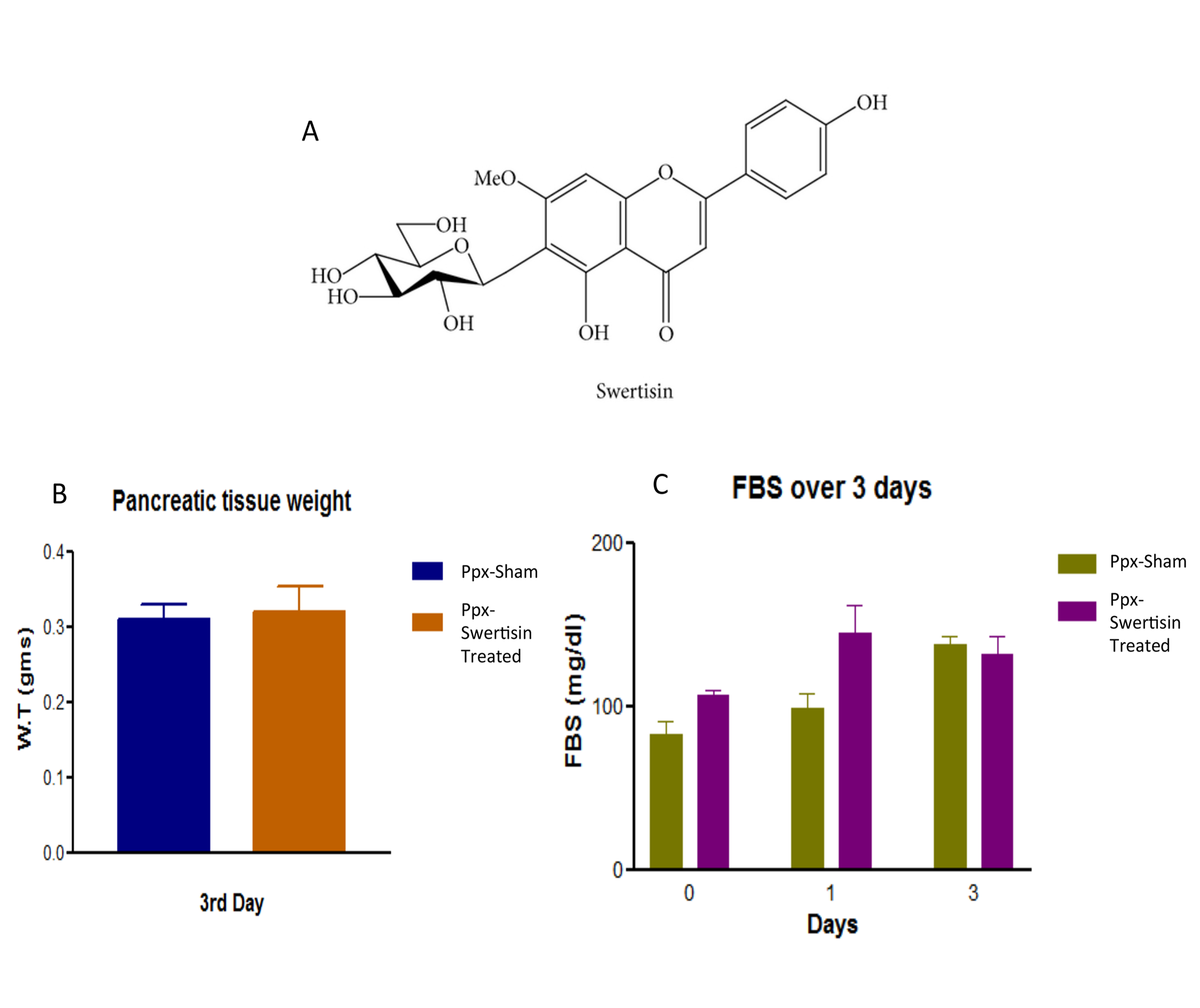

Supplement: S2 Fig — (Figure A) showing Swertisin molecular structure. (Figure B) graphs demonstrate change in pancreas weight calculated on 3rd day post sacrifice and (Figure C) shows fasting blood glucose in sham operated and Swertisin treated animals. (TIF) [file pone.0128244.s002.tif]

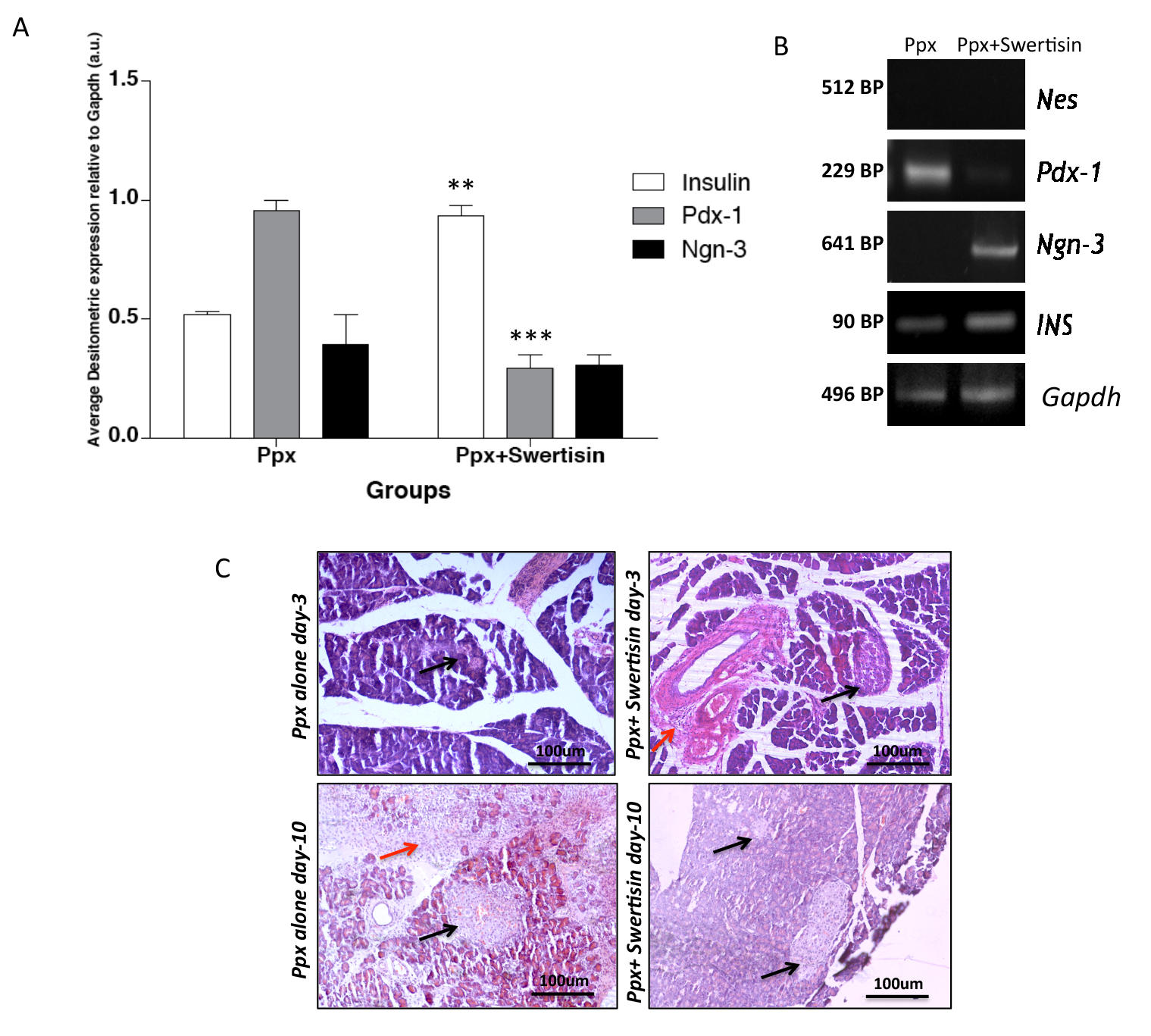

Supplement: S3 Fig — (Figure A-B) shows gene expression data for various islet specific markers in these regenerating pancreatic tissues. Quantitate mRNA expression of genes expressed in post Ppx with and without Swertisin treatment was analyzed. All data seta are represented as mean ± SEM and calculated from 3 independent animal observations. *** and ** represents p value < 0.001 and 0.01 Vs Ppx animals. (Figure C) shows images of pancreas regeneration at 10 day, demonstrating beta cell regeneration (TIF) [file pone.0128244.s003.tif]

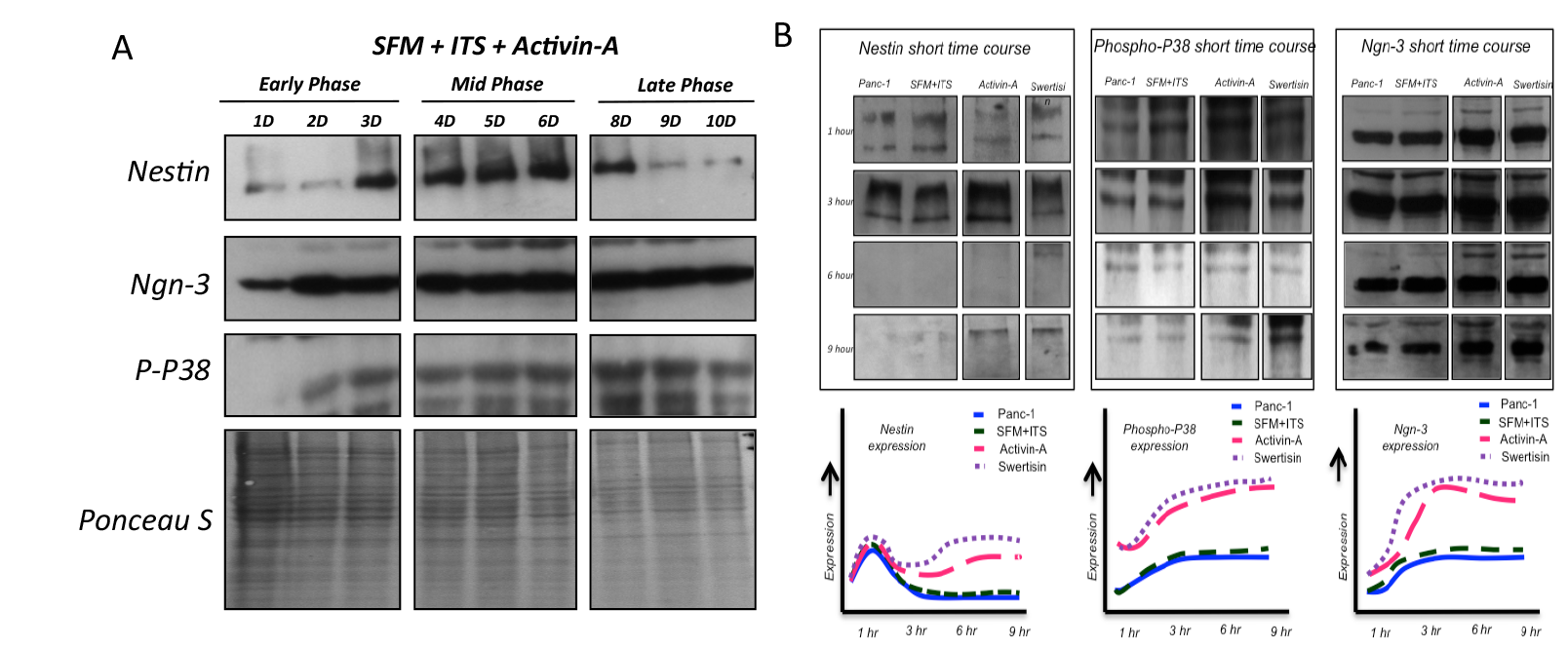

Supplement: S4 Fig — (Figure A) shows western blot profile of activin-A mediated islet differentiation in panc-1 ILCC in time dependent manner. (Figure B) shows protein profile of key differentiation markers in short time 0–9 hours. (TIF) [file pone.0128244.s004.tif]
