# Supplementary material for: Swertisin an Anti-Diabetic Compound Facilitate Islet Neogenesis from Pancreatic Stem/Progenitor Cells via p-38 MAP Kinase-SMAD Pathway: An In-Vitro and In-Vivo Study
Source: PLoS One. 2015 Jun 5;10(6):e0128244. doi: 10.1371/journal.pone.0128244 (PMC4457488; doi:10.1371/journal.pone.0128244)
Supplement: S1 Table — Shows list of primary antibodies used in ICC, IHC and western Blot experiments with particular details for each experiment like specificity, dilution factor, molecular weight etc. (DOCX) [file pone.0128244.s005.docx]

| **Name of**  **Antibody** | **Company** | **Target against**  **species** | **Mono/**  **Polyclonal** | **Mol. Weight**  **(kDa)** | **Source** | **Dilution for**  **WB** | **Dilution for ICC** |
| --- | --- | --- | --- | --- | --- | --- | --- |
| C-peptide | Cell signalling | Hu, Rt, Mo | Poly | 4 | Rabbit | NA | 1:100 |
| Insulin | Cell signalling | Hu, Rt, Mo | Poly | 6 | Rabbit | NA | 1:100 |
| Glucagon | Sigma | Hu, Rt, Mo | Mono | 3.5 | Mouse | NA | 1:200 |
| Beta Actin | BD | Hu, Rt, Mo | Mono | 42 | Mouse | 1:5000 | NA |
| Ki 67 | Sigma Aldrich |  | Mono | 345/395 | Mouse | 1:2000 | 1:400 |
| Nestin | Sigma Aldrich | Hu, Rt, Mo | Mono | 177 | Rabbit | 1:1000 | 1:250 |
| Pdx1 | Cell Signaling | Hu, Rt, Mo | Mono | 42 | Rabbit | 1:1000 | 1:100 |
| Ngn-3 | Santa Cruz | Hu, Rt, Mo | Mono | 27 | Mouse | 1:1000 | 1:100 |
| Phospho-P-38MAPK | Cell Signaling | Hu, Rt, Mo, Mk, Sc | Mono | 43 | Mouse | 1:2000 | 1:400 |
| P-38MAPK | Cell Signaling | Hu, Rt, Mo, Mk, Gp | Poly | 43 | Rabbit | 1:1000 | 1:200 |
| E-Cadherin | BD | Hu, Rt, Mo, Dg | Mono | 120 | Mouse | 1:2500 | 1:250 |
| N-Cadherin | SantaCruz | Hu, Mo, Rt | Poly | 140 | Rabbit | 1:1000 | NA |
| Vimentin | Sigma Aldrich | Hu, Rt, Mo | Mono | 53 | Mouse | 1:1000 | 1/250 |
| Smad-2/3 | Cell Signaling | Hu, Rt, Mo, Mk | Mono | 60/52 | Rabbit | 1:1000 | NA |
| Smad 7 | R & D Systems | Hu, Rt, Mo | Mono | 50 | Mouse | 1:500 | 1:50 |
| Ck-19 | Sigma Aldrich | Hu, Rt, Mo, Mk, Gp | Mono | 40 | Mouse | 1:1000 | 1:100 |
| PARP-1 | Cell signalling | Hu, Rt, Mo, Mk | Mono | 116 | Rabbit | 1:1000 | 1:400 |
| Caspase-3 | Thermo Scientific | Hu, Rt, Mo, Rb | Poly | 32/17 | Rabbit | !:500 | NA |
| Phosho-Smad-2 | Cell signalling | Hu, Rt, Mo, Mi | Mono | 60 | Rabbit | 1:1000 | 1:50 |

**Supporting information Table S1**

**Table S1: List of Antibodies used in western and ICC/IHC**
