# Supplementary material for: Swertisin an Anti-Diabetic Compound Facilitate Islet Neogenesis from Pancreatic Stem/Progenitor Cells via p-38 MAP Kinase-SMAD Pathway: An In-Vitro and In-Vivo Study
Source: PLoS One. 2015 Jun 5;10(6):e0128244. doi: 10.1371/journal.pone.0128244 (PMC4457488; doi:10.1371/journal.pone.0128244)
Supplement: S2 Table — Shows list of forward and reverse primer sequences on all genes used in RT-PCR experiments along with melting point and amplicon size for each gene. (DOCX) [file pone.0128244.s006.docx]

**Supporting information Table S2**

**Table S2: List of primer sequences used in RT-PCR**

| **Name of Gene** | **Gene Accession**  **Number** | **Primer Sequence Forward** | **Primer Sequence Reverse** | **PCR Conditions**  **(Tm)** | **Amplicon**  **Size (BP)** |
| --- | --- | --- | --- | --- | --- |
| Nestin | [NM_016701.3](http://www.ncbi.nlm.nih.gov/nucleotide/50363231?report=genbank&log$=nucltop&blast_rank=3&RID=J5A8KR9S014) | GCGGGGCGGTGCGTGACTAC | AGGCAAGGGGGAAGAGAAGGATGT | 58 | 512 |
| PDX-1 | [NM_022852.3](http://www.ncbi.nlm.nih.gov/nucleotide/50838801?report=genbank&log$=nucltop&blast_rank=1&RID=J5A2TA9X014) | CTC GCT GGG AAC GCT GGA ACA | GCT TTG GTG GAT TTC ATCCAC GG | 55 | 229 |
| Ngn-3 | [NM_009719.6](http://www.ncbi.nlm.nih.gov/nucleotide/157267375?report=genbank&log$=nucltop&blast_rank=1&RID=J59ZDAYK015) | ACTAGGATGGCGCCTCATCCCTTG | GGTCTCTTCACAAGAAGTCTGAGA | 57 | 641 |
| Insulin | NM_008386.3 | GCCCAGGCTTTTGTCAAACA | CTCCCCACACACCAGGTAGAG | 55 | 90 |
| GAPDH | [NM_001289726.1](http://www.ncbi.nlm.nih.gov/nucleotide/576080554?report=genbank&log$=nucltop&blast_rank=14&RID=J59VDR3A015) | CAAGGTCATCCATGACAACTTTG | GTCCACCACCCTGTTGCTGTAG | 58 | 496 |
